# Covalence in Cow-veillance: Sensing Technologies and Human-Animal Affinities in Dairying

**DOI:** 10.1177/01622439241280177

**Published:** 2024-09-27

**Authors:** Camille Bellet, Emily Kathryn Morgan

**Affiliations:** 1 Centre for the History of Science, Technology and Medicine, 5292 University of Manchester, Manchester M13 9PL, UK; 2 Department of Art and Visual Culture, 1177 Iowa State University, Ames, IA 50011, USA

**Keywords:** human-animal relations, surveillance cameras, remote sensing, farming, affinities, care

## Abstract

This article considers how the widespread use of camera surveillance systems in dairy farming affects engagements between farmers and cows. While literatures on visual surveillance often cast monitoring technologies as cold and mechanical tools for enabling and reinforcing hegemonic power relationships, our research leads us to question this reductive formulation. Drawing on ethnographic research with dairy farmers, as well as archival materials from agricultural presses in the United Kingdom and France, we argue that applying camera surveillance on dairy farms extends emotional investments and affinities between farmers and cows. Challenging dominant critiques of visual surveillance, we consider cows as subjects and not just objects of the camera gaze and bring attention to the sensorial experiences of farmers relying on cameras as vital tools. We explore how experiencing visual images of cows, particularly real-time images displayed on computers and smartphones, engenders new sensations among farmers; and we speculate about how cows’ own material realities and sensory experiences of being farmed may change with the advent of remote-sensing cameras. Our study offers an innovative way of looking at human-animal relationships in agriculture, one focused on the evocative and sensorial experiences of remote visual monitoring, from human and nonhuman animal perspectives.

In early 1983, two articles heralding the arrival of camera surveillance systems on dairy farms appeared within just a couple of months of one another in a pair of popular farming magazines. *Farmers Weekly*, an English-language periodical published in Britain, and the French-language *Agromatique*, each included an article focused on the use of TV cameras in cow barns. The two publications cast the development as an unmitigated good, both for the farmers profiled in the articles and for the cows they observed. *Farmers Weekly* itself sponsored the adoption of the equipment on one English farm, and its article “TV ‘Eye’ on the Pen” quotes a farmer as saying, “This is pure convenience for me” (Jones and Barker 1983, 71). Early in his career, the farmer, John Batchelar, kept his calving pen outdoors and close to his house, with his cows calving virtually underneath his bedroom window, all during a late-spring calving season. But the process of increasing production on his farm and industrializing his output led to an earlier and longer calving season, during which cows calved indoors, in a barn far distant from Batchelar's house. Keeping an eye on calving meant long walks across the farmyard, often in cold night-time weather. With the adoption of the TV cameras, the article notes, Batchelar can again supervise calving “from the relative comfort of the bedroom,” now through a TV monitor (Jones and Barker 1983, 71). Likewise, the French article notes that with a camera surveillance system, farmers would be able to set up an alarm, “a beep, beep under the pillow,”^1^ allowing them to take a quick look at the TV monitor every two or three hours without leaving their beds (Meliet 1983, 50). Profit, too, increases with the system. A second farmer quoted in the British article, George Barker, notes that surveillance of his herd has yielded a decrease in calf mortality, an important benefit as costs of siring his Holsteins have gone up. The surveillance system “more than pays for itself if you manage to save a couple of calves,” Barker asserts (Jones and Barker 1983, 71).

Images included within the British article reinforce these narratives of convenience and profit: the caption of one image describes “Bedside Batchelar” viewing his cows and controlling the camera angle with a remote control while sitting on his bed; while another image depicts cows on a TV screen, the camera hardly appearing to interfere with the animals’ regular barn lives. A third image depicts Barker in his barn, pointing at his camera while a cow stands behind him, placid and undisturbed, her muzzle affectionately close to his ear (Figure 1). The French article, written by a civil servant and functioning more like promotion than independent journalistic exploration, likewise emphasizes the proximity of surveillance, enterprise, and profit: “Could the surveillance camera [already used in] department stores, S.N.C.F. railway stations, hotels, etc. not be used in agriculture?” the author notes (Meliet 1983, 50). The image accompanying the article, depicting a swiveling closed-circuit camera, reinforces this notion of the ubiquitous, all-seeing camera eye.

**Figure 1.**
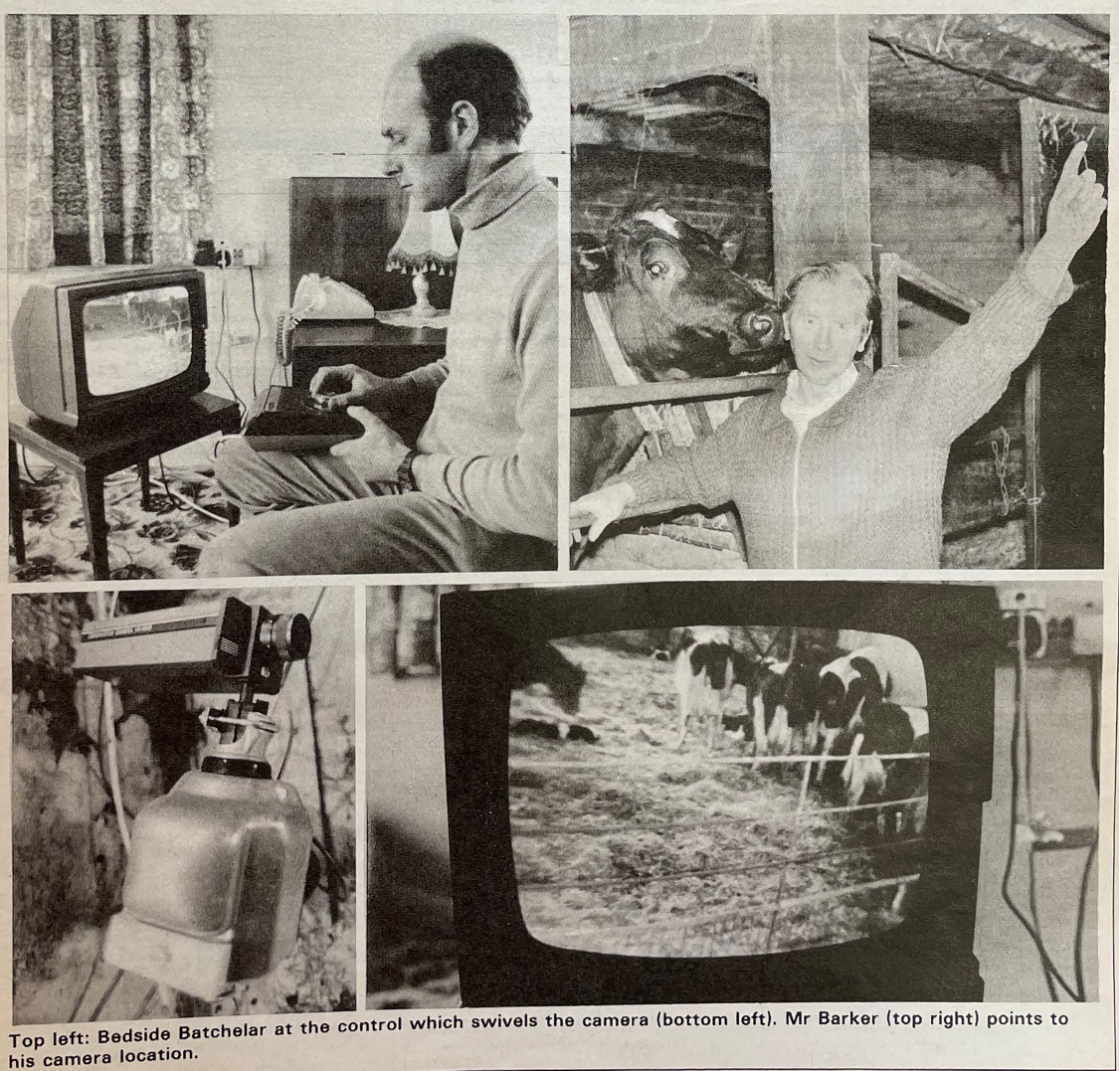
“TV ‘eye’ on the pen,” *Farmers Weekly*, January 28, 1983. Collection of the Museum of English Rural Life (MERL), United Kingdom.

The systems described in these early-1980s articles as novelties are now, in the 2020s, extremely common sights on farms. Farmers deploy cameras and other sensing systems in a wide variety of configurations, to perform a wide variety of tasks: monitoring herd behavior, checking the condition of barns and equipment, managing birthing. Literature describing, explaining, and promoting such visual sensing systems abounds. But discourse about the advantages of installing these systems has changed little since the 1980s: emphasis still lies on the benefits of herd surveillance for convenience and profitability. Little attention has focused on the complexities of farm surveillance, and there has been almost no discussion in scholarly literature of how remote visual monitoring has affected relationships between farmers and their animals, or how the “material realities” (Hawkins and Dibley 2021, 695) of animals themselves might change under farming surveillance.

This article attempts to correct these omissions, addressing farming surveillance systems’ capacities to show cow worlds and to reconfigure human–animal relationships in dairy farming. Our study pays particular attention to the sensorial elements of day-to-day data collection on farms using remote visual monitoring. Sociologist and STS scholar Andrew Balmer (2021, 1159) has argued that reorienting one's approach to data, paying attention to the aesthetic and sensorial nature of information, may help one to see “the shapes, forms and moulds that make us, our relationships and our worlds.” Building on this idea, we consider how encounters not only with cows, but with real-time camera images of cows displayed on computers and smartphones, change farmers’ experiences of and with cows.

Combining ethnographic methods with methods drawn from feminist studies, STS, sensory studies, and visual culture studies, this interdisciplinary study illuminates the more-than-optical aspects of remote digital imaging surveillance in animal agriculture. The study draws on qualitative interviews with fifteen farmers (four women and eleven men), as well as hundreds of hours of ethnographic observations on five dairy farms in France and the United Kingdom (UK) between 2022 and 2023. The farms averaged 200 dairy cows, with the smallest housing 70 cows and the largest 1,500. Some farms used manual milking methods; others had milking robots. Some respondents were technicians employed by the farm, others were herd managers, and still others owned the farms. All respondents worked full-time on the farm, spent at least half their day in person with the cows, and had access to the barn cameras on a phone or laptop.

Observations took place over a week on each farm. Days began around 5 AM and ended around 5–6 PM; with additional nocturnal visits lasting about two hours from 7–8 PM daily or, on some farms, every other day. On three farms, coauthor Bellet also spent time in the farmer's house. All participants were informed of the research and their right to withdraw participation at any time. They were given assurances about anonymity, and ethical approval was given by Bellet's institution. Fieldwork notes and interview transcripts were analyzed inductively (Charmaz 2006). Data from observations and interviews were complemented with analyses of archival published written and visual accounts and promotions of farm cameras and sensing technologies in major French and British agricultural presses from the late 1970s onward.

We argue that camera surveillance systems on dairy farms, marketed to farmers as a means of increasing profitability and convenience, also affect their relationships with cows. Filmic connections to cows strengthen human–animal *covalence—*attachment and connection*—*on the farm. They may also change or even deepen farmers’ emotional investment in cows. Dairy cows’ sensory experiences of being farmed, too, change with the advent of remote-sensing cameras. While it is impossible to gauge cows’ psychological experiences, the article finds that remote sensing cameras bring real changes in how animals are housed and moved, how and when they are disturbed or physically affected during calving, even whether they are awakened from sleep. While the panoptic qualities and hegemonic effects of animal surveillance certainly merit evaluation, this study contends that the subtleties of farmer–cow relationships as mediated through cameras, the *covalence* that results from *cow-veillance*, also warrant nuanced examination. By cow-veillance, we do not only refer to human surveillance of cows from above, as suggested by the prefix “sur,” on. We also contend that by letting cows alone physically, the camera affords cows greater self-regulation and promotes their agency*—*what Mann (2002) calls their *sousveillance*, from the prefix “sous,” below.

## Visual Surveillance, Control, and Care

Vast and burgeoning STS literatures address the mechanisms of surveillance, its justifications, its growth, and its ubiquity (see e.g., Jensen and Draffan 2004; Hildebrand 2021). Foremost among these are mechanisms of vision: tools, structures, and processes that enable humans to extend and augment the powers of the eye to produce new knowledges, orders, and realities. In Western societies, human sight has traditionally been framed as more authoritative and masculine than other “primitive” or “feminine” senses, such as smell and touch (Haraway 1991; Howes and Classen 2014). Extending the capabilities and the authority of human sight—what film scholar Anat Pick calls the “male/humanist gaze” (2015a)—the camera becomes, symbolically and sometimes literally, a powerful machine eye itself: cold, neutral, and systematic, a brainless eye that sees for seeing's sake. As a tool for marginalizing the subject and reinforcing human power structures and social orders, the camera eye has been unparalleled.

Numerous works in media, visual, and film studies have addressed with eloquence the visual dimensions of disciplinary structures (e.g., Maurer 2017; for an overview, see Guido 2019). Building on Jeremy Bentham's model architecture of reformist surveillance, the Panopticon, Michel Foucault (1975) analyzes how entire Western societies assimilate this self-policing impulse, watching themselves (and watching themselves watch themselves). The most canonical surveillance camera in Western literature, in George Orwell (1949)'s *1984*, peers into every corner of every home, seeing every action of every person, perpetuating and extending the absolute power and panoptic vision of the state. Every person becomes an observed individual, a coded figure—a “dividual” (Deleuze 1992): deformable, transformable, and dependent on the goodwill of the state.

In farming, animals are kept and raised for business and production gain. Everything about their bodies and their lives—from what they eat, to where they sleep, to how they move and reproduce—becomes subject to human control through the datafication of their bodies and lived experiences. Cameras are easily assimilated to the overall machinery of agricultural control to increase traceability and productivity: tools right at home among the branding irons, tail dockers, ear taggers, and monitoring devices. We do not dispute the panoptic nature or the “scopic regime” (Maurer 2017) of visual surveillance technologies in farming, which we have also seen at work in field observations. We do, however, argue that there is more at stake with the use of farm cameras than sight and surveillance, solely for the purposes of control and domination. In disrupting this narrative, we hope to foreground individual experiences typically excluded from the current industrial model of camera surveillance in animal farming.

In looking at the everyday presence, the “vernacular material life” (Ott, Serlin and Mihm 2002, 2), of cameras in dairy farming, we argue that another way of sensing, engaging, and experiencing the cow with and through the camera eye, is unfolding daily and routinely on farms. In farming surveillance systems, the camera eye *monitors* the target animal: checking, watching, *keeping an eye on* the animal or the herd, as a parent or caretaker does when monitoring a child in a crib or bed (on child monitoring technologies, see Bennett 2001). And just as with parental surveillance, farmer surveillance is driven by more-than-optical senses and sensations. Farm surveillance functions as a multisensory experience that weds the farmer more closely to the animal.

“The photograph's power derives as much from its affective magic as from its realist claims, and ultimately from the powerful combination of the two” (Cvetkovich 2014, 276). Cross-modal activation of sight with other senses when viewing an image can transform fields of photographic vision (Howes 2010). Seeing an image while hearing, smelling, touching, and tasting may open an observer to unpredictable sensations, emotions, and perceptions (Fijn 2019; Palmer 2021), ultimately challenging the notion of the farm camera as an exclusively cold eye, useful for maintaining a distant care.

Through an app on a phone or an image on a laptop, the farmer carries the animal in a pocket or case. The farmer takes animals into the kitchen or living room, checks on them, monitors their noises in the night from the warmth of the bedroom, transports them—and the care of them—to the pub, to restaurants and shops, even on holiday. A farmer watching a live feed of a happy, placid cow while drinking and listening to music at a cozy pub; or checking on a cow from the comfort of a living room, full of family and homey scents, might feel a sense of openness, camaraderie, and sympathy with the animal. A farmer checking on a laboring cow while waiting in a cold, sanitized doctor's office, on the other hand, might experience a heightened sense of anxiety and uncertainty, the animal's bodily exertions and vulnerabilities calling the farmer's own embodied experience into sharper focus. Conversely, images of a happy, placid cow might calm an anxious farmer, while farmers monitoring a laboring cow while having a pint might find the hoped-for relaxation out of reach. These mediated multisensory experiences, situated in varied spaces with their own unspoken standards, have real and powerful effects, influencing the ways farmers understand, live with, and care for cows. This, in turn, changes cows’ realities too. Such profound affective developments, we argue, warrant careful consideration. Indeed, as Bennett (2001, 208) argues, cameras, like many other sensing technologies, are not “merely aids to human activity, but also powerful forces acting to reshape that activity and its meaning” (see also Mann 2002; Ellis, Tucker and Harper 2013; Agostinho, Maurer and Veel 2020).

Several studies have examined how publicly available webcam live-streams can transform human experiences and ethical obligations toward domesticated, feral, and wild animals (e.g., Benson 2010; Kamphof 2013; Pick 2015b). However, very few empirical studies have focused on cameras’ effects on relations between humans and farmed animals. Farmed animals such as cows are often framed as uncharismatic and overly commodified, and monitoring of animals by farmers has generally been presented as utilitarian and instrumentalist (Arcari, Probyn-Rapsey and Singer 2021). In discussions about animal surveillance, questions of farmer–cow sensibility or affection rarely come into play. But the everyday, “ordinary” (Stewart 2007), cinematic practices of seeing cows through a lens and transporting them through different spaces with a camera can be powerfully multisensory and emotionally complex for farmers. The varied sensations made possible by the camera's observation of the cow give what Stewart (2007, 2) calls affective “circuits and flows” to everyday farming experiences.

Stewart describes “ordinary affects” as the constant, core sensations that shape individual experience, guiding one's actions (including toward others) and sometimes generating emotions such as compassion, anxiety, or nostalgia (Stewart 2007, 2). Extending Stewart's argument, we find that ordinary affects become inflected by the camera's distinctive way of mediating cow bodies, times, and spaces. Farmers’ ordinary affects, we propose, change with the camera, shaping their feelings, actions, and care toward cows. The use of visual technologies on farms and the mediation of farmer–cow relationships by cameras do not imply the negation or total breakdown of traditional interspecies care relationships. On the contrary, our study suggests that the increasing use of cameras in cow barns drives an intensification of already existing practices and covalences—what Jennifer Mason (2018) terms “affinities”—between farmers and cows. Camera surveillance on farms also stimulates the construction of new realities in cow care, in which time, space, and technology intertwine.

## Careless/Caring Vision: the Dairy Industry's Scopic Regime

Since the 1970s and 1980s, pressures on farmers have increased rapidly. The growth of the cattle industry has generated widespread complications, including increased herd sizes, concentration of calving, and labor shortages. Faced with a surfeit of animals and a dearth of assistance, farmers have come to depend on cameras as vital prosthetic eyes, supplementing their own and augmenting their powers to monitor their herds. As others have noted (see e.g., Fineman 1999; Jain 1999), the camera is a powerful tool for retaining mastery over an ever-expanding industrial and productivist infrastructure. From the 1970s, articles in the farm presses, like the ones discussed at the outset of this article, have promoted camera technologies as harbingers of a new era in farming. Such sources generally cast the camera eye as reliable, highly efficient, and neutral, with its “viewing system which enables you to see *exactly* what you are going to get” (Dixon 1981a, 103, our emphasis). Some early articles on farm cameras promoted them as “a satisfying, even profitable, hobby” (Dixon 1981a, 103), but this quickly changed, cameras becoming not a form of entertainment but a vital technology, permitting farmers to keep close, sustained visual contact with their herds. Like the eye of God, one article promises, the camera allows farmers “to be everywhere at once” (Meliet 1983, 50).

Published as a seven-part series, “Photography on the Farm” appeared in the British farming magazine *Farmers Weekly* in 1980–81. Throughout the series, author Barry Dixon promotes the panoptic vision afforded by farm cameras, offering pointers to farmers on how to best photograph their fields, their cows, their buildings, and their machinery. Much of Dixon's advice centers on photographs made for purposes of documentation: photographs as evidence of ownership, for instance. But some discussion, and some images, hint at the use of cameras as permanent fixtures of the farm, installed in barns for full-time monitoring of facilities and herds. One image depicts a rotary milking parlor, photographed with a “fish-eye” lens. Dixon's (1981b, 94) text claims that such lenses, with their all-seeing but radically distorting view of the barn interior, would be unusual: the image, he notes, was made by the *Farmers Weekly* staff photographer, not by a farmer, and “such lenses are expensive and properly belong in the realm of the specialist professional photographer.”

**Figure 2.**
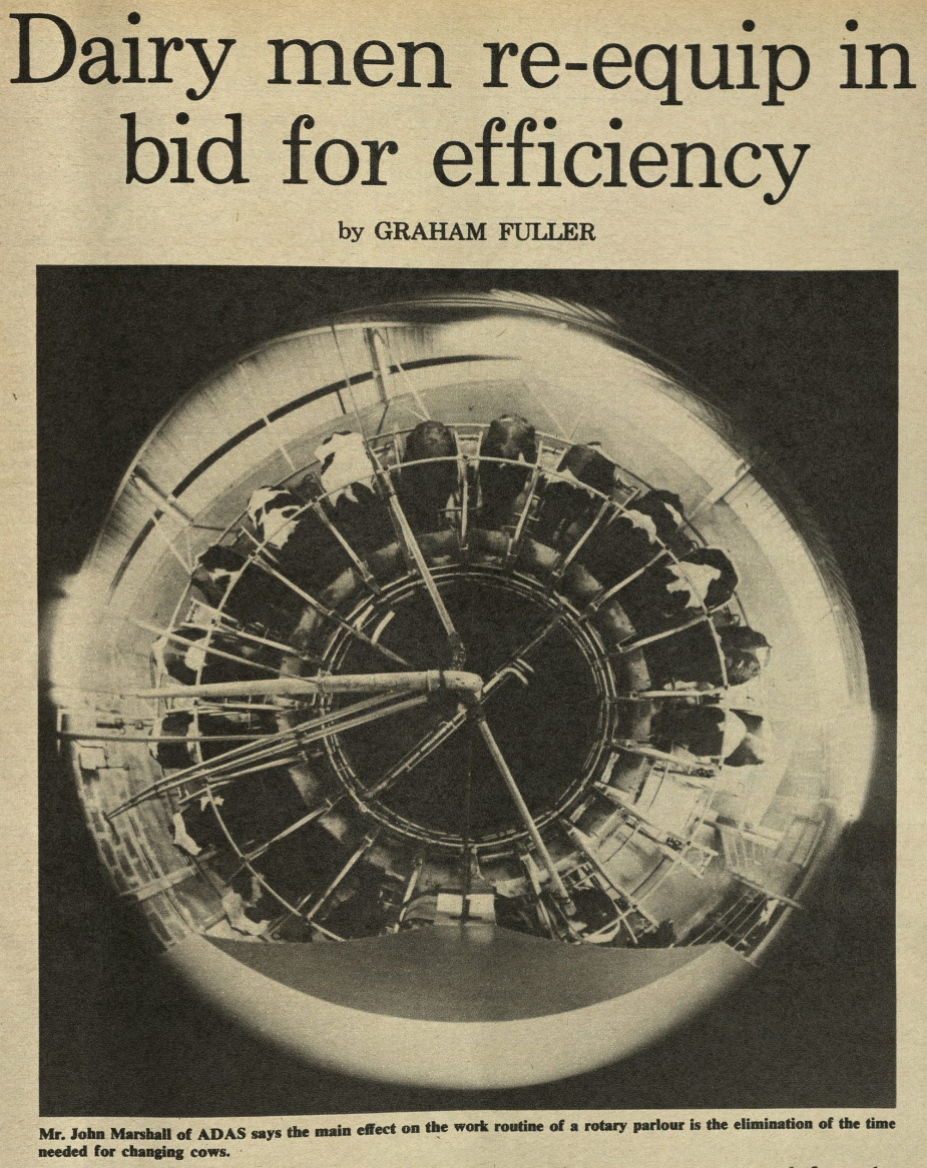
“Dairy men re-equip in bid for efficiency,” *Farmers Weekly*, February 8, 1980. Collection of the Museum of English Rural Life (MERL), United Kingdom.

But another *Farmers Weekly* article by Graham Fuller (1980), “Dairy Men Re-Equip for Efficiency,” shows that fish-eye lenses were already installed in some facilities.^2^ The image on the first page depicts a fish-eye-lens view of a rotary milking parlor (Figure 2). Installed directly on the ceiling of the milking parlor, above the center of the feeder, the lens affords a vision of the rotary milking platform as a pie chart, each cow constituting a very profitable slice, their bodies almost visually merging with the center of the farmer's machine. The cows, virtually linked to the machine by “collar-borne transponder[s]” (Fuller 1980, xxviii), become physically linked by the lens too. This top-down vision of cows as integral components of the machine reinforces the productivist and patriarchal industrial trajectory of animal farming. Through the cold-fish gaze of the fish-eye lens, the “dairy men” of the title view every cow body as a production unit: each identifiable by a “three-digit” radio transmitter code (Fuller 1980, xxviii), each a single, living “dividual” (Deleuze 1992, 7) of the overall milk production machinery. Such overview visions of the milking herd, produced by dome-shaped barn cameras, remain quite common sights on dairy farms, affording farmers constant, comprehensive views.

The commodification of cows’ bodies, specifically of their reproductive functions, lies at the heart of the dairy industry. To make milk, cows must first become pregnant and give birth. The milk their bodies create is then diverted into human food systems. Many feminist scholars have drawn parallels between the commodification of cow productivity and reproductivity, and the subjugation and instrumentalization of human women's bodies and reproductive functions under capitalist patriarchy (e.g., Adams 1990). In these and many other *Farmers Weekly* articles, cameras appear as exclusively male attributes, tools wielded solely by men to supervise female reproductive processes.^3^ Both of the British farmers interviewed for the 1983 *Farmers Weekly* article discussed at the start of this study explicitly address the benefits of camera surveillance in supervising birthing. Since dairy farming pivots on controlled calving, monitoring of pregnancy and birthing are central to the business model. As one respondent farmer, David,^4^ running a farm of around 1,500 cows in the UK puts it, “the majority of cameras are for cows… [to] look [at cows] …see if they’re calving, if they need any help, or if they’ve calved.”

Today, the importance attached to camera surveillance is even higher, and is complemented by burgeoning use of other sensing, recording, and data-collecting equipment for monitoring cow breeding. Linked to CCTV or accessible and controllable via smartphone app, cameras contribute visual information to vast constellations of data. Inserted in a pierced ear, an ear tag sensor transmits biometric information. Mounted on the leg, head, or neck, other sensors offer information about movement rates and/or patterns. Devices monitor estrous cycles, milk production, and body conformation; and in pregnant cows, they predict calving. Some devices of the latter type, such as the Agrimonitor, might be mounted around the abdomen or elsewhere on the body to detect contractions or changes in temperature indicating impending birth; others such as the Vel’Phone would be inserted transvaginally. Cameras constitute points for the collection of visual data, to join the wealth of other data collected by this variety of cow sensors (Grodkowski et al. 2018).

Crucially, however, there is a disconnect between how visual technologies are promoted by the dairy industry and how farmers actually use them. Whereas the industry has tended to regard the power of farmer–cow emotional bonds as unworthy of remark, many farmers regard their connections with and affection for cows as a core feature of their work. Whereas the dairy industry promotes remote sensing as a means of increasing profits, farmers tend to think of the value of camera monitoring for the safety and security of cows. Farmers often regard cow birthing as an event where less, rather than more, intervention is preferable—a preference with which farm cameras prove quite compatible. Unless the birth is complicated, it is generally safer for both cow and calf if the farmer does not intervene. Farm cameras have, increasingly, permitted this passive oversight.

Farmers report that cameras facilitate oversight of a birth experience that is literally less intrusive than other forms of monitoring. The camera “means you don’t get involved so much, which is always the best thing,” one farmer, Jeremy, emphasizes. Cameras today really increase the farmer's ability to see the birth remotely, while letting the cow alone, physically. “I can turn [the camera] on from the computer. It's exceptional when I have to get up,” explains Corinne, another farmer, who routinely monitors her 70 cows from her own bed. As another respondent, François, explains, “cows can also calve outside and I have a camera right here at the top [of this post], hanging there, which is used for calving, at night, on weekends,” or at other times when he is away. François “just check[s] on the cameras… [to] see if [the calving] is progressing…if the calf is there.” If he sees that the calving “is complicated, that the legs [of the calf] are not in the right direction,” he goes to the cow barn to intervene and assist, to serve as doula and midwife to the cow.^5^ But this is not the usual situation. Usually, the cow tends to her own birth. The modern, prosthetic camera super-eye magnifies the farmer's powers of observation, allowing for the practice of a less invasive form of care.

Surveillance cameras are often lumped together, in dairy industry literature, with other sensing devices—part of broader monitoring systems. This characterization is often accurate, to be sure. But farmers themselves often drew a distinction between the camera and other sensing devices they had tried. The intravaginal Vel’Phone probe, designed explicitly for monitoring cow pregnancy, deploys a spider-like cluster of sensors to collect data about rising body temperature, intensification of contractions, and other signals of impending labor.^6^ Yet many farmers attempting to use the device disliked it intensely. They reported that the cow often ejects the device before or during labor, rendering it useless and requiring the farmer to root around for it in piles of manure. Inserting it could be dangerous for the farmer: more than one reported resisting the kicks of unhappy, probe-averse cows. Farmer Corinne's resistance to the device was perhaps the strongest of all, and was grounded not in her concerns for herself, but for the cow: she considered the probe disruptive, uncomfortable, even “disgusting” for the cow; and she worried about the risk of infection.

The Vel’Phone probe and other data-collection devices attempt to supplant entirely the farmer's powers of observation and replace them with data, a replacement that is often rejected (both by the cow *and* by the farmer!) as unnecessary and irritating—an unwelcome intrusion of science, technology, and the clinical into a space traditionally occupied by the cow herself, assisted by the farmer. Cameras, on the other hand, complemented and extended farmers’ own observational powers, permitting them to see cows’ behavior without influencing it. As Corinne explains, “I always look at the camera before [going to the barn] …I have the camera on my laptop at home and I watch them. Because the cows are used to…hearing the car, the doors, they know. So, I look beforehand” so as not to disturb them. Such affective data contrasts with many dairy industry reports on farm cameras and augmented-farming technology, where the augmented farmer is almost universally presented as an authoritatively benevolent Big Brother (Edwards Murphy et al. 2015), not as a bonded, *covalent* carer. These reports generally emphasize augmented farming's benefits for industrial agriculture, noting how such systems monitor and transmit cows’ data to farmers’ computers and smartphones 24 hours a day (e.g., Grodkowski et al. 2018; Torres 2019). They offer no real evaluation of the transformative, sensorial experiences brought by digital technologies, or of their implications for farmers, cows, and their relationships.

## The Digital Portable Cow

**Figure 3.**
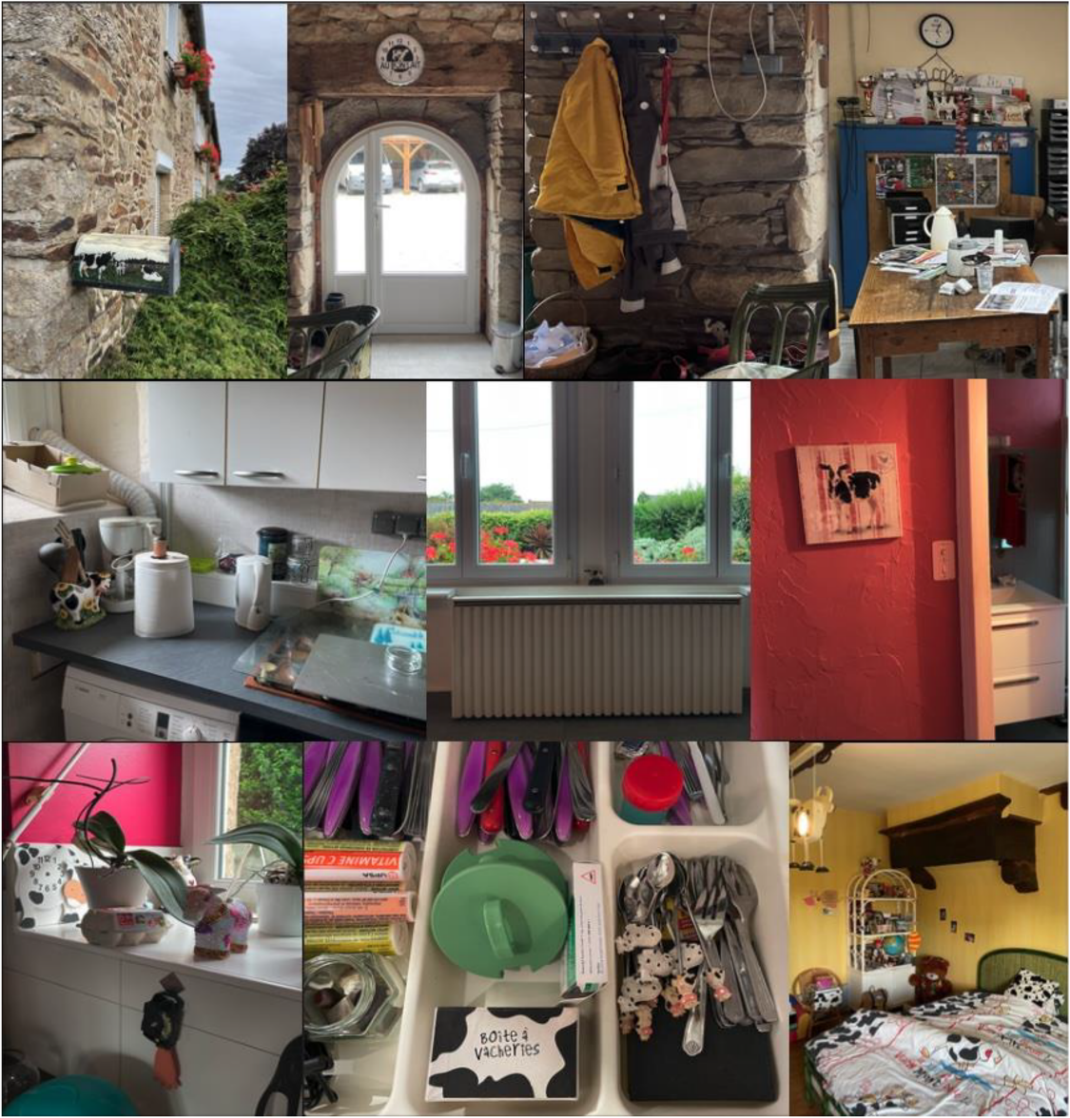
Cow-themed household goods in one farmer's household, 2021–23. Credit: Camille Bellet.

Through camera interfaces, farmers carry their herds with them wherever they go. The phone slides into a pocket, and with it goes the entire herd, in miniature. In some ways, the digital, pocket-sized cow merely extends some farmers’ existing proclivities: many dairy farmers collect cow memorabilia and cow-themed household goods. Such items—pictures, clocks, utensils, etc.—may be seen to extend farmers’ authority over their herd, their ability to possess and control cows, both literally and symbolically (Figure 3). Making the huge animals miniature, cute, precious, and decorative strips them of any real presence or force. “The cleverer I am at miniaturizing the world,” writes Gaston Bachelard (1994, 150), “the better I possess it.”

**Figure 4.**
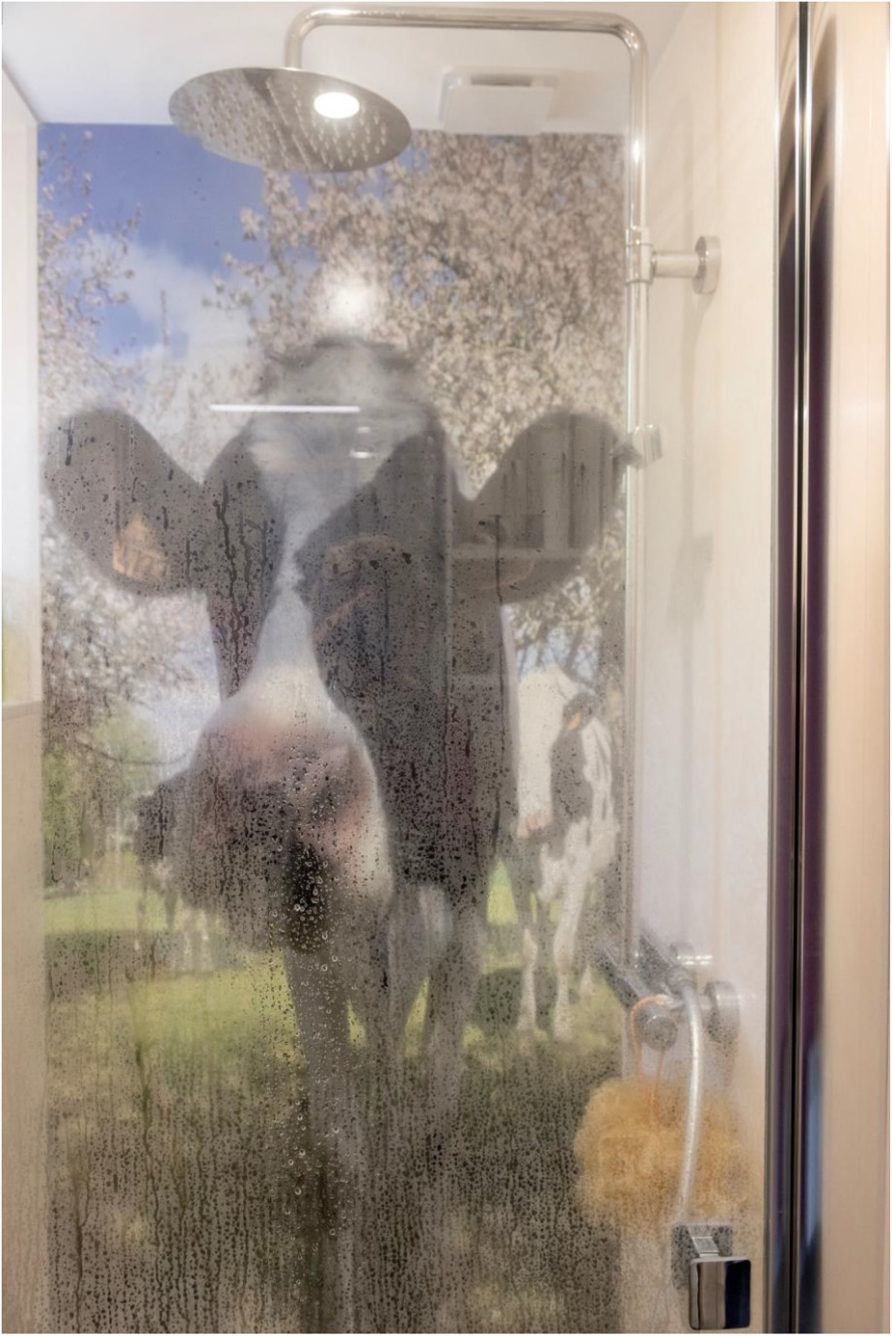
Showering with Paros: a photograph mounted on the back wall of the shower stall in one farmer's home, June 6, 2023. Credit: Liz Hingley.

But, Bachelard (1994, 150) continues, “it must be understood that values become condensed and enriched in miniature.” Cow trinkets and miniatures commemorate and aestheticize farmers’ companionship and caring relationships with their herd, serving as talismanic symbols of their affection and regard for cows. From the back garden to the bedroom and in between, miniaturized cows lurk everywhere in some farmers’ houses, framing every space and time of the farmer's day (Figure 3). Cows are present for some farmers even in moments of deepest vulnerability, such as while naked in the shower (Figure 4).

**Figure 5.**
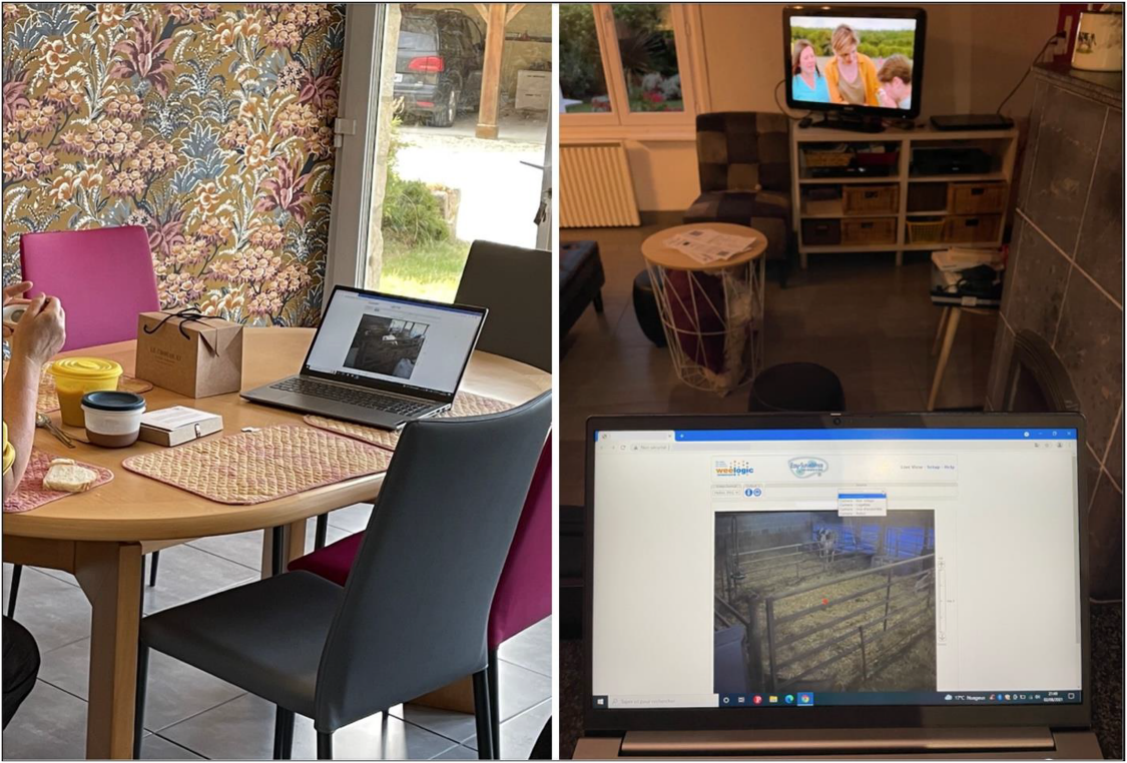
Left: Breakfasting while waiting for Résidence's baby, June 6, 2023. Right: Waiting for Orssay's baby, August 2, 2021. Credit: Camille Bellet.

The miniaturized, digital cow becomes a talisman too: an effigy of the real cow, through which the farmer implements understanding, care, and affection for the living animal. Indeed, while the cow miniature or trinket seems inert, the digital portable cow represents the real cow, transported and integrated into the farmer's household space. The miniaturized digital cow moves across the computer screen, a real-time animal counterpart to the farmer's family members (Figure 5, left) or the actors on the television playing in the background. As Corinne monitors a laboring cow, Orssay, from her kitchen one evening, the exciting tension of Orssay's impending birth supersedes that of the televised drama (Figure 5, right). The digital cow participates in family conversations and household activities.^7^ Teletransported by the camera, the miniature cow infiltrates the house, a space not freely accessible to her full-sized, fleshly body. She can breathe, eat, and drink in the kitchen; get milked in the living room; and give birth in the farmer's bedroom.

The farmer's experience of the miniaturized digital cow is transformative too. Often it is pleasant, as the smells of fried onions and hot soup, or physical contact with freshly laundered bedding, replace sensations associated with manure, dust, and humidity. For Corinne, a living presence brings comfort and even emotional support as she lies alone in her bed at night: she explains how the miniaturized digital cow by her side distracts her from the shadows and heavy silence of the night, caused by the absence of her late husband. The camera makes it possible for her to sleep beside her cows as often as she wishes.^8^ For another farmer, Wallace, the affection given and received by the cow is a help, almost a refuge from the many difficulties, frustrations, and resentments of daily life in farming. As Wallace puts it: “Sometimes when you’ve had a tough day or you’re upset, looking at them and getting close to them makes you feel like [all this hard work] is worth it.”

But for other respondents the distilled emotions prompted by the miniaturized digital cow are not so enjoyable: François’ wife feels annoyance and frustration at the constant presence of the digital cow. The moving miniature amplifies an already too-present feeling of overflow, linked to her husband's inability to disconnect from his work at the end of the day. What her husband (and promoters of farm cameras more broadly) see as convenience for assisted calving from the bedside, François’ wife experiences as intrusion—digital technology rendering the cows inescapable.^9^

Cameras also extend farmers’ sensory capabilities. “With digital zooming…you can really see what's going on at the back end of a cow who is standing at the far end of the shed,” Jeremy explains. The imperceptible, dark scenes of the cow's life become visible through technology. In addition, the camera eye “automatically adjusts according to the light available and, at night, switches to infrared,” François notes. With her hazy edges and soft focus, the nocturnal, infrared cow invites the farmer to connect differently. Sitting on the sofa or lying in bed, in the warmth of the home, the farmer is no longer strained by the cold and humidity of the night in the barn, and is no longer constrained by the limitations of the unaided eye. Farmer Max explains that he can rest with cows from the tumult of the day, physically removed but virtually and imaginatively linked to the cows’ own experiences of the night. The darkness itself “creates a sense of solidarity” (Pallasmaa 2005, 48).

**Figure 6.**
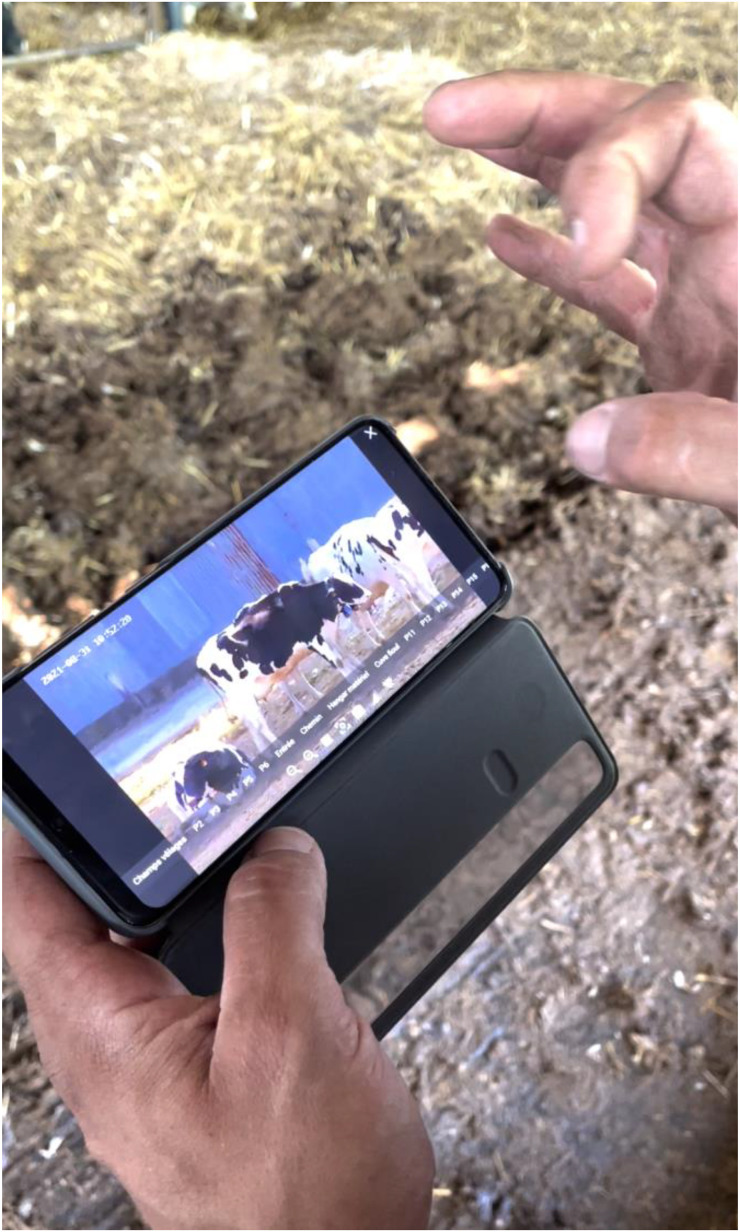
Spinning the cow, video still, August 31, 2021. Credit: Camille Bellet.

Looking at miniature paintings, Hanneke Grootenboer (2012, 42) argues that it is not only the space and the time in which a miniature image is situated, “but the [miniature] object as such that designates a place of intimacy.” Through the farm camera, the cow becomes the object. Unlike a miniature painting, the digital portable cow retains her dynamism too—not only in the sense of her own mobility but in a new sense, enabled by digital tools. Rather than “static” (as farmer Steve noted about older camera systems), the digital cow spins, slides, or hurtles across the screen as the farmer pans, tilts, or zooms the camera eye (Figure 6), thus forming a new relational system of vital relations.

**Figure 7.**
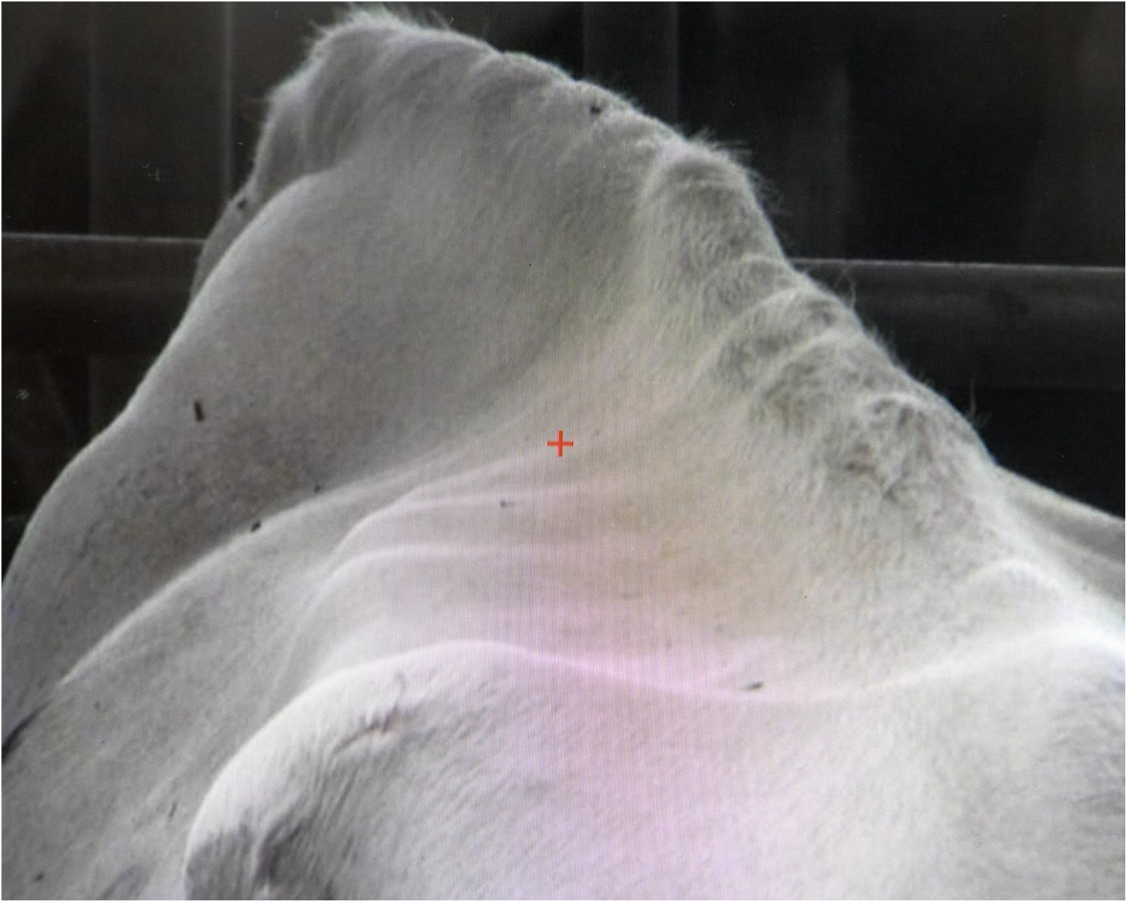
Magnifying the cow, June 6, 2023. Credit: Liz Hingley.

“Zooming is very easy and the pixelation is good too,” François asserts. Sophisticated camera technologies facilitate closeness: just as botanists access and touch the “intimacy of flowers” with the aid of their magnifying glasses (Bachelard 1994, 153), farmers embrace the body of the cow with their camera and their screen, engaging in “haptic viewing” (Kamphof 2013). They approach, pinch, and flick the different parts of her body, shrinking or enlarging them at will, zooming to inspect the cow down to “the smallest detail of every one of her hairs,” as another farmer, Katherine, notes (Figure 7). By soliciting and rewarding farmers’ tactile engagements, the digital screen multiplies the ways one observes a cow (Fay 2008, 51). Zooming enhances the stature of the bovine subject: “The enlargement of a snapshot does not simply render more precise what in any case was visible, though unclear,” Walter Benjamin ([1935] 1969, 236) wrote, “It reveals entirely new structural formations of the subject.”

## Plunging into the Cow Longue Durée

To watch cows is to recognize their distinct experience of the world, one profoundly different from the human. Large, slow, and ponderous, cows live in a state of sameness, a *longue durée* that most humans in Western societies would find unbearably protracted, unbearably boring (Macgregor Wise 2004). Documentary filmmaker Emmanuel Gras's 2013 film, “Bovines ou la vraie vie des vaches” (Bovines or the real life of cows), allowed viewers to look at cows (*just* cows) for more than an hour, from a cow's-eye angle. Making visible processes that are too slow for human time-speed, Gras opened the cow world to his audience, shifting and adjusting it to cow life (see Pick 2015b). Gras aimed to convey how an apparent void, constituted by a repetition of banal activities like browsing, rumination, and sleep, might constitute a full-fledged existence; and how slow cinema could convey the value of that existence to a non-farming audience.

But many dairy farmers already know these things. For them, live-streaming cows is a daily activity, complementing and expanding their experiences of the physical animal. Live-streaming cows requires farmers to follow “cow time,” to watch at a cow's pace, even to adopt, to some extent, a bovine sense of the passage of time.^10^ For the farmer, this can be an attractive idea—a perk of the job. “It's super interesting to watch the cows moving. I have always loved that,” Corinne reports.

Footage from farm cameras, too, may spark viewers’ imagination, “prompt new modes of looking, seeing” (Pick 2015a), new modes of empathetic identification with cows, and new cow “subjectifications” (Macgregor Wise 2004, 425). Indeed, farm cameras’ capacity to do so may even exceed what cinema can do, because the farm camera exists without the constraints typically placed on commercial culture. No producer forces it to justify its existence by turning a profit or generating buzz; it conforms to no narrative arc. The farm camera's audience is limited: the farmer and perhaps a small circle of family, visitors, and workers. In installing the system, the farmer establishes a hyperlocal cinema, relevant and suited to the conditions of the farm itself and prioritizing the needs of the cow above all other narratives. On the farm, CCTV cameras prioritize and reinforce cow time, aestheticizing the everyday rhythmic routines of the nonhuman animal subjects whose bodies and interests lie at the heart of dairy farming.

Writing of popular cinema, Benjamin ([1935] 1969, 234) argued that “distraction and concentration form polar opposites” in experiences of the filmic image, with cinema seeming to reward a casual, distracted viewership different from the absorbed viewership of the traditional art connoisseur. But the dairy farmer-viewer's caring eye functions, in some ways, like a cinematic connoisseurship, a total absorption, through the image, in bovine realities and bovine time: nondualism enabled by CCTV. By capturing “images which escape natural vision” (Benjamin [1935] 1969, 220), the camera eye draws farmers into an alternate, bovine world. The camera permits a break with the hectic ordinary daily life of dairy farming, “downplay[ing] event in favor of mood, evocativeness” (Jonathan Romney, cited in Çaglayan 2016, 64).

The camera eye brings a new vision of the cow “unattainable to the naked eye yet accessible to the lens” (Benjamin [1935] 1969, 220). Observing cow sensations, imagining cow dreams, one may begin to inaugurate new cow realities. We can imagine a cow differently, more empathetically, by scrutinizing her and observing the continuous and slow effects of time and light on her movements and body. One “who reads slowly and examines images in slow motion, lingering as long as is needed over each image,” Bachelard (1994, 152) notes, “will experience a sort of coalescence of unlimited values.” Prolonged attention, enlargement and slow motion absorb farmers into bovine existence, exacerbating their already-present tendency to plunge into cow realities. Rather than having “to sit on a bale of straw to meditate and think about the cow,” as Corinne noted, it is now possible for farmers to do so from the palm of their hand. Anytime and anywhere, a farmer can pause and, as Bachelard (1994, 159) said of miniatures more broadly, “take the time needed to see all these little things that cannot be seen all together.” Watching the digital portable cow, the farmer accedes to new forms of digital reverie.

## Digital Reverie as Resistance to the Scopic Regime of the Dairy Industry

Our study has attempted to correct what we perceive as a lack of attention to affective, sensory, and aesthetic dimensions of farmers’ engagements with cows through farm cameras. We refer to the aesthetic not—or not only—in the sense of beauty, but in the sense of a reflective and contemplative engagement with the image, and thus with what the image represents, i.e., the cow herself. Often, the notion of connoisseurship, of deep knowledge derived from close aesthetic engagement, carries with it connotations of elitism; but it can just as easily describe more everyday engagements. Dairy farmers already engage in what might be considered cow connoisseurship—an engagement with cow bodies, cow appearances, and cow lives far different and far deeper than that of the average human individual. Indeed, their success as farmers and animal caretakers relies on this connoisseurship of cow-ness: on constant close looking, close listening, close smelling, close engagement with all the senses. The sensory input derived from these connoisseurial engagements, and the decisions they make based on what they sense, constitute the very essence of farming. Aesthetic experience is not a luxury but, we contend, crucial to daily farming practices. We have attempted to show how farm cameras and digital imaging technologies extend farmers’ aesthetic experiences, reinforcing and deepening their sensorial engagement, bonding, and care for cows.

In considering farm cameras, we found notions of the camera eye as exclusively cold, exclusively panoptic, to be limited, unsatisfying, and inaccurate to characterize lived and observed experiences of contemporary farming. Monitoring cows through the camera seems, more often, a productive, generative activity, allowing human viewers to attend to and think anew about cow realities in farming. Farmers care for cows with their barn cameras, tending to the daily business of the farm. But the cameras also create space for contemplation, for introspection, for speculation. Farm cameras and digital imaging technologies begin to admit a cow-centered vision, a cow-centered reality. They permit human viewers to begin to understand what it might be like to see like a cow, even if it is not fully possible to understand that experience.

Cows themselves, of course, see their own worlds in a way humans cannot imagine. As Yong (2022, 72 emphasis in the original) has pointed out:

Cows…don’t need to [turn their heads to look at you]. Their visual fields wrap almost all the way around their heads…giving them a view of the entire horizon at once….A cow can simultaneously see a farmer approaching [her] from the front, a collie walking up from behind, and the herdmates at [her] side.

The farm camera cannot permit the farmer to see like the cow, much less to exist like her; total interspecies identification is beyond any of us. But it can allow us to begin to imagine what it might be like to breach our own boundaries, to go beyond the limitations of human vision. Writing of how “slow cinema” encourages an alteration in the viewer's perception, Emre Çaglayan (2016, 74 emphasis added) refers to a “*ruminative* mode of spectatorship:” the slow, attentive spectator seeing, thinking, and perceiving like a cow. Pick (2015a, 108) notes that such contemplation may “reconfigur[e] the connections between visuality and ethics in favour of animals.” Cameras have already allowed us to see microscopically, to see into space, to see into the interiors of bodies (and walls, and suitcases) (Moholy-Nagy 1936). Why shouldn’t they allow us to begin to see, if not exactly like a cow, then at least with greater respect for her, greater appreciation for her lived experience?

In his book *The Opening of Vision: Nihilism and the Postmodern Situation*, David Michael Levin (1988, 439) differentiates two types of gaze: One “imposes on people a conformity to predetermined representation,” while the other is grounded in caring that “can encourage others to be true to themselves, so that they may develop their ownmost potentialities.” A modern, patriarchal “observation,” Levin continues, supposes that objects maintain coherence and are stable in their relations to one another over time; that looking happens instantaneously and passively; and that the observer neither affects nor is affected by what is observed. “Contemplation,” by contrast, involves “a sense of wonder and enchantment” (Levin 1988, 101). The contemplative viewer engages in looking as an activity conducted over a duration, and *is acted upon* by what they contemplate.

Contemplating the cow, the farmer-viewer enters a state separate from the workaday mode of thinking about the day-to-day preoccupations of dairy farming. Contemplation, for the farmer, may even become a mode of resistance to industrial agriculture, a means of bringing the farmer in line with cow time and cow existence, rather than the other way around. Resting the eyes and the psyche with (and in) the cow, the farmer disregards and disrupts the rhythms of intensive farming, acceding instead to bovine time. Cowveillance has the capacity to shift farmer-cow covalences, displacing the productive intensity of cow rearing, replacing it at least in part with an increased sense of farmer–cow affinity.

## Notes

1. All translations of French sources were made by Camille Bellet.

2. Other photographs in *Farmers Weekly* from around the same time show that fish-eye lenses were increasingly common on farms, not only the realm of the “specialist professional photographer,” as the Dixon article claims, but an already-integral part of farmers’ tools and their overall agricultural machinery.

3. In the *Farmers Weekly* series “Photography on the Farm,” photography is exclusively connected to masculinity. There are many examples where the photographer is portrayed as a bold and adventurous male character. If a female appears, she is there to assist and remind us of the combative effort of the male photographer who literally crawls on the ground to reach his target (e.g. “Photography on the Farm,” Part 3, *Farmers Weekly*, January 30, 1981, 94).

4. Throughout the text, the names of human participants have been replaced by pseudonyms. Names of cow participants have remained original, at farmers’ request.

5. During one of coauthor Bellet’s farm visits, two farmers, a married couple, noticed that a cow they were monitoring appeared to be having a difficult calving. The pair rushed to the barn and called the veterinarian. When the vet arrived, the male farmer stood by the vet, holding the cow by the tail, and helping the vet properly reposition the calf and pull the calf out of the cow, while the female farmer crouched in front of the cow, stroking her muzzle and reassuring her.

6. A photograph of the device is available at: https://www.medria.fr/fileadmin/Medria/Medias/Version_anglaise/Fiche_Vel_Phone_GB.pdf [Accessed July 6, 2024].

7. While preparing dinner and chatting with her daughter, Corinne watches her cows through the camera. “These are my babies,” she says. Corinne explains how the camera allows her to watch the cows, to know how they are. She found this especially important, if a bit stressful, on a day when visiting technicians were resurfacing the cows’ walking and sleeping areas, requiring the herd to be confined in an area smaller than normal. “I don’t like seeing them all stuck together and huddled like this,” she noted while looking at the camera image. “I prefer to watch them [moving around].”

8. Corinne’s late husband used to tease her about this ever-present attention and emotional self-projection toward their cows, saying to her: “you know you can sleep [in the barn] with them if you want.”

9. Interestingly, Corinne embraced both cow trinkets—which proliferated in her house—and the digital cow; whereas François’ wife, who was uncomfortable with the constant presence of filmed cows in her house, likewise had few cow-themed objects about.

10. As on “most live Internet camera sites,” on cow CCTV cameras “precisely *nothing* happens most of the time.” (Palmer, cited in Macgregor Wise 2004, 428, emphasis original). As with Warhol’s slow tasting of a fast-food hamburger in his film, *Eating a Hamburger* (1982) (see Boczkowska 2020), the slow viewing of cows’ life through CCTV cameras opposes the rhythms of mainstream commercial dairy production, pointing to new forms of visual aesthetics.
